# Quantitative nodal burden as a predictive marker for chemotherapy benefit in postoperative ampullary adenocarcinoma: a multi-institution population-based study

**DOI:** 10.3389/fimmu.2025.1610334

**Published:** 2025-09-19

**Authors:** Zheng Li, Chongyuan Sun, Zefeng Li, He Fei, Cheng Xing, Dongbing Zhao

**Affiliations:** ^1^ Department of Pancreatic and Gastric Surgical Oncology, National Cancer Center/National Clinical Research for Cancer/Cancer Hospital, Chinese Academy of Medical Sciences and Peking Union Medical College, Beijing, China; ^2^ Department of General Surgery, Beijing Hospital, National Center of Gerontology, Institute of Geriatric Medicine, Chinese Academy of Medical Sciences, Beijing, China

**Keywords:** ampullary adenocarcinoma, adjuvant chemotherapy, LODDS, lymph node burden, chemotherapy benefit, survival

## Abstract

**Background:**

The effectiveness of adjuvant chemotherapy (ACT) in ampullary adenocarcinoma (AA) patients and under what conditions ACT should be applied are not clearly defined.

**Methods:**

The study encompassed a series of consecutive AA patients who underwent curative surgical resection. These patients were sourced from the National Cancer Center of China, spanning the years 1998 to 2020, as well as from 12 registries within the Western surveillance, epidemiology, and end results program, covering the period from 2004 to 2017. The quantitative nodal burden was evaluated using the log odds of positive lymph nodes (LODDS). The correlation between ACT and overall survival (OS), recurrence-free survival (RFS), and cancer-specific survival (CSS), was meticulously evaluated using Cox proportional hazards regression models and tested by interaction analysis.

**Results:**

Despite its rarity, a total of 948 and 225 eligible patients were included in the Western cohort and the China cohort, respectively. ACT was not significantly associated with improved long-term survival of unselected AA patients in both the China cohort (OS: *P* = 0.86, RFS: *P* = 0.84) and Western cohort (OS: *P* = 0.11, CSS: *P* = 0.82). After the quantitative analysis of nodal tumor burden, the study revealed that in patient subgroup with LODDS exceeding -1.4, significantly prolonged survival outcome emerged in patients received ACT (Surgery+ACT *VS.* Surgery alone, HR: 0.38, 95% CI: 0.19 - 0.75, *P* = 0.01). The multivariate analysis demonstrated that increasing LODDS was associated with increasing survival benefit from ACT (*P* for interaction = 0.03), whereas the N classification did not reliably identify patients benefiting from ACT based on statistical test of interaction (*P* = 0.20).

**Conclusion:**

This study emphasizes the potential of LODDS to inform personalized therapeutic decisions in the management of AA.

## 1 Introduction

Although the rare onset of ampullary adenocarcinoma (AA) accounts for only 0.2% of gastrointestinal malignancies, the incidence has been escalating annually at a rate ranging from 0.5% to 0.9% since 1970 (1). To date, surgical resection remains the only potentially curative method for AA (2). Compared to adenocarcinomas of the adjacent pancreatic duct, common bile duct, or duodenum, AA is associated with a more optimistic prognosis (3, 4). However, poor rates of long-term prognosis through surgery alone necessitate a multimodality strategy for it (5, 6).

According to National Comprehensive Cancer Network (NCCN) guidelines, adjuvant chemotherapy (ACT) can be used in all stages of AA. The ESPAC-3 trial demonstrated that among postoperative AA patients, ACT was not associated with a significantly improved survival in the primary analysis; after adjusting for prognostic factors, multivariable analysis showed a statistically significant survival benefit associated with ACT (7). Although several reports showed the benefit of adjuvant therapy, existing studies also indicated the opposite result (8). A multicenter study suggested that no difference in long-term survival was observed between ACT and surgery alone; however, the survival benefit was found in patients with pT4 or pN1-2 (9). Due to the shortage of evidence, no consensus on indications for the use of ACT has been reached in relative guidelines, including NCCN, Japanese Society of Hepato-Biliary-Pancreatic Surgery (JSHBPS) (10), and European Society for Medical Oncology (ESMO) (11).

Regarding the status of lymph node burden, despite the widespread use of N-classification, its predictive utility in stratifying patients for ACT is limited by its qualitative nature. Quantitative methodologies, such as log odds of positive lymph nodes (LODDS), have been prioritized in recent research to resolve these deficiencies (12), which may be useful in identifying patients benefiting from ACT. Given these, the study collected experiences from China and the West to determine if long-term survival outcome after curative resection of AA could be improved by the selection of patients for ACT by LODDS.

## 2 Method

### 2.1 Patients and chemotherapy regimens

In this international, retrospective cohort study, the cohort of Chinese enrolled patients who underwent curative surgery for AA at the National Cancer Center of China, from January 1, 1998, to December 31, 2020 (China cohort). The Ethics Committee of the hospital approved the study. Informed consent was secured from participants, authorizing the utilization for potential research. Additionally, patients with AA between January 1, 2004, and December 31, 2017, in the surveillance, epidemiology and end results program were collected as the Western cohort. In which, 12 registries were enrolled including Alaska Natives, California, Connecticut, Georgia, Hawaii, Iowa, Kentucky, Louisiana, New Jersey, New Mexico, Seattle (Puget Sound), and Utah. The median follow-up time of the China cohort and Western cohort was 35 months (ranges: 1~217 months) and 34 months (ranges: 1~188 months), respectively. Additionally, power analysis was implemented to ascertain the appropriate sample size. The study followed the Strengthening the Reporting of Observational Studies in Epidemiology (STROBE) reporting guidelines (13). The study was approved by the National Cancer Center of China ethics committee (NCT06435767). Written informed consent for participation was collected from all patients.

Pathologically confirmed AA patients without distant metastasis treated with curative-intent resection were included in the study. According to the exclusion criteria, patients who received neoadjuvant therapy or radiotherapy, as well as those with missing clinical pathological or follow-up information, were excluded. Variables including patient sex, age, pathological characteristics, postoperative complication, hematological data, and treatment modalities were collected from the medical records. Histologic subtype of AA was not routinely determined in the Hospital from 1998 to 2020 and the pathological report did not necessarily include immunohistochemical staining features. Lymphovascular invasion (LVI) was defined by the presence of neoplastic cells within lymphatic spaces and muscular vessels. Pathologic staging (TNM) was classified based on the American Joint Committee on Cancer Staging Manual (AJCC, 8th edition).

In our center, postoperative chemotherapy regimens and doses were planned by oncologists, with adjustments made based on drug toxicities or tumor responses. ACT regimens for participants included 8 3-week cycles of GS (1000 mg/m^2^ of gemcitabine intravenously on day 1; 8 and 60 mg/m^2^ of S1 orally on days 1 to 14), 4 3-week cycles of GX (1000 mg/m^2^ of gemcitabine intravenously on day 1; 8; 15 and 830 mg/m^2^ of capecitabine orally on days 1 to 21), 4 3-week cycles of GEMOX (1000 mg/m^2^ of gemcitabine as a 30-minute intravenous infusion on day 1; 8 and 130 mg/m^2^ of oxaliplatin as a 2-hour intravenous infusion on day 1), 6 3-week cycles of GP (1000 mg/m^2^ of gemcitabine intravenously on day 1; 8 and 75 mg/m^2^ of cisplatin intravenously on days 1 to 3), 6 3-week cycles of XELOX (130 mg/m^2^ of oxaliplatin as a 2-hour intravenous infusion on day 1 and 1000 mg/m^2^ of capecitabine orally on days 1 to 21), 6 3-week cycles of SOX (130 mg/m^2^ of oxaliplatin as a 2-hour intravenous infusion on day 1 and 60 mg/m^2^ of S1 orally twice a day on days 1 to 14), 6 2-week cycles of FOLFOX4 (85 mg/m2 of oxaliplatin intravenously on day 1, 200 mg/m2 of folinic acid as a 2-hour intravenous infusion followed by a 400-mg/m2 bolus of fluorouracil, and a 22-hour intravenous infusion of 600 mg/m2 of fluorouracil), and 6 3-week cycles of single S1 (60 mg/m^2^ of S1 orally twice daily on days 1 to 14).

The primary outcome, overall survival (OS), was defined as the time elapsed from the date of surgery to either the date of death or the last contact. Recurrence-free survival (RFS) was defined as the duration from the surgery date to the occurrence of recurrence, last contact, or death unrelated to recurrent malignancy. Cancer-specific survival (CSS) was defined as the interval between the date of surgery and the date of death due to AA or last contact.

### 2.2 Statistical analysis

Descriptive statistics were provided as frequencies with percentages for categorical variables and median with interquartile range (IQR) for continuous variables. Factors associated with the use of ACT were identified by steady Poisson regression adjusted by the sandwich variance estimator. We conducted a distribution test and selected parametric and nonparametric statistical methods based on whether they followed a normal distribution. The distribution of continuous variables was examined using the t test or Wilcoxon rank sum teat, as appropriate. Pearson χ^2^ test was used to test the distribution of categorical variables. Estimates of survival rates were calculated using the Kaplan-Meier method, and the distributions of survival were compared between groups by log-rank test. Univariate and multivariate Cox proportional hazard models were applied to find the prognostic factors of long-term survival, including OS, RFS, and CSS. Variables with a univariate P-value < 0.10 were included in the multivariate model to ensure a robust selection of prognostic factors. Correlation coefficient was used to examine the multicollinearity of factors and backward selection was performed. The LODDS was defined by formula:


$$LODDS=lg\frac{Number\;of\;N^{+}+0.05}{Number\;of\;N^{total}-Number\;of\;N^{+}+0.05}$$

N^+^ and N^total^ refer to positive lymph node and total examined lymph node, respectively. LODDS was chosen for its capacity to reflect the overall lymph node burden and to account for node-negative patients. For each specific value of LODDS, a hazard ratio (HR) was calculated by comparing survival between patients with LODDS greater than that number (LODDS > i) and those with fewer than or equal to that number (LODDS ≤ i). Then, the adjusted HR for benefit analysis was calculated using the formula:


$$adjusted\;HR_{i}=\frac{cHR_{i}}{nHR_{i}}$$

cHRi and nHRi refer to the HR (when LODDS = i) in patients treated with chemotherapy and non-chemotherapy, respectively. Adjusted HR for each LODDS was summarized in **Supplementary Table**. For ease of understanding, taking LODDS = -1 as an example, among patients who received chemotherapy, the cHR for those with LODDS greater than -1 compared to those with LODDS less than or equal to -1 was 0.4. In contrast, among patients who did not receive chemotherapy, the nHR for those with LODDS greater than -1 compared to those with LODDS less than or equal to -1 was 1.1. Therefore, at this point, the adjusted HR is calculated as 0.4/1.1 = 0.36. LOESS regression was used to establish a smooth model and the Spearman test was performed to identify the correlation across each point. Significance levels were defined as two-sided *P* < 0.05. All statistical analyses were conducted using R software, version 4.2.0 (R for Windows) and IBM SPSS software, version 25.0 (IBM Corp). Data analysis was conducted from February 1, 2024, to April 18, 2024.

## 3 Result

### 3.1 Baseline characteristics

**Figure 1:**
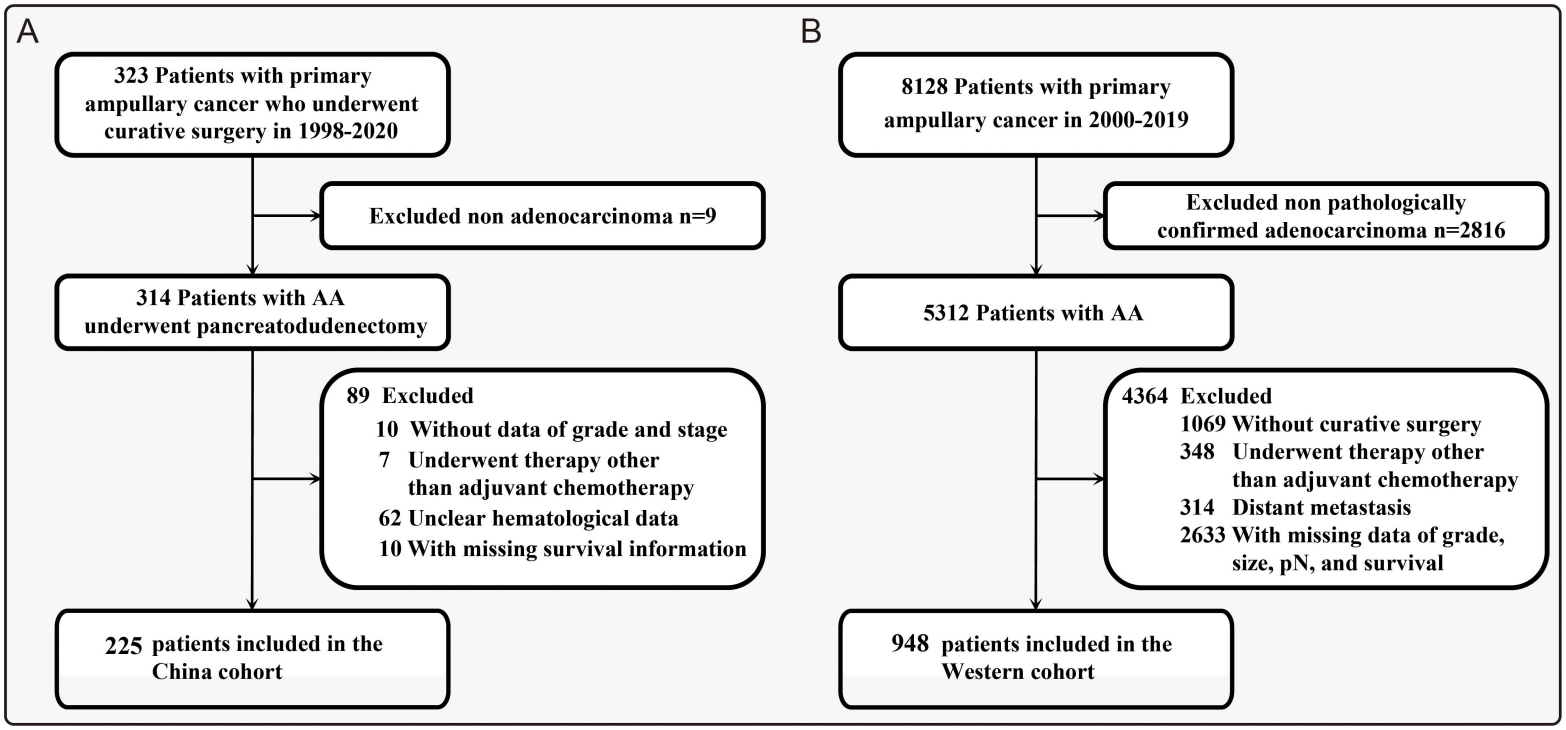
The progress of patient enrollment of this international cohort study. **(A)** Flowchart of patients from a high-volume tertiary hospital in China (China cohort). **(B)** Flowchart of patients within the Western surveillance, epidemiology, and end results program (Western cohort).

In the China cohort, a total of 323 patients with ampullary adenocarcinoma undergoing curative resection were screened in the studied period. After the exclusion of patients with other pathological types (n = 9), missing pathological data (n = 10), adjuvant therapy other than ACT (n = 7), missing hematological data (n = 62), and incomplete follow-up information (n = 10), 225 patients (mean [SD] age, 56.8 [10.2] years, 132 males [58.7%]) met the inclusion criteria (**Figure 1A**). Compared with patients without ACT, patients treated with ACT were more likely to be younger (<65 years, 169 [75.1%] *vs* 56 [24.9%]; relative risk [RR], 4.67, 3.55, and 3.36 for <45, 45-55, and 55–65 years, respectively), with higher T stage (T3-4, 122 [54.2%] *vs* 103 [45.8%]; RR, 2.53; 95% CI, 1.39-4.61), with positive lymph node (70 [31.1%] *vs* 155 [68.9%]; RR, 2.08 and 2.77 for N1 and N2, respectively), poor differentiated (90 [40.0%] *vs* 135 [60.0%]; RR, 2.10; 95%CI, 1.26-3.49), and with LVI (59 [26.2%] *vs* 166 [73.8%]; RR, 2.19; 95%CI, 1.35-3.56) (**Table 1**).

**Table 1:** Demographic, clinicopathologic, and outcome characteristics.

Variable	Value (N = 225)	RR (95%CI)
Sex, n (%)		
Male	132 (58.7%)	1 [Reference]
Female	93 (41.3%)	1.31 (0.79-2.15)
Age, n (%)		
<45	27 (12.0%)	4.67 (1.58-13.81)
45-55	67 (29.8%)	3.55 (1.27-9.95)
55-65	75 (33.3%)	3.36 (1.20-9.38)
≥65	56 (24.9%)	1 [Reference]
AJCC 8th pT, n (%)		
T1	33 (14.7%)	1 [Reference]
T2	70 (31.1%)	
T3	117 (52.0%)	2.53 (1.39-4.61)
T4	5 (2.2%)	
AJCC 8th pN, n (%)		
N0	155 (68.9%)	1 [Reference]
N1	56 (24.9%)	2.08 (1.22-3.52)
N2	14 (6.2%)	2.77 (1.36-5.62)
AJCC 8th TNM stage, n (%)		
I	89 (39.6%)	1 [Reference]
II	94 (41.8%)	4.058 (1.879-8.763)
III	42 (18.7%)	3.330 (1.390-7.978)
Size, median [IQR]	2.20 [1.5, 3.0]	1.10 (0.89-1.36)
Grade, n (%)		
Poorly differentiated; Grade III	90 (40.0%)	2.10 (1.26-3.49)
Moderately differentiated; Grade II	91 (40.4%)	1 [Reference]
Well differentiated; Grade I	44 (19.6%)	
Lymphovascular invasion, n (%)		
No	166 (73.8%)	1 [Reference]
Yes	59 (26.2%)	2.19 (1.35-3.56)
Chemotherapy, n (%)		
No/Unknown	177 (78.7%)	NA
Yes	48 (21.3%)	
Complication, n (%)		
No	130 (57.8%)	1 [Reference]
Yes	95 (42.2%)	1.06 (0.64-1.76)
Blood transfusion, n (%)		
No	112 (49.8%)	1 [Reference]
Yes	113 (50.2%)	1.08 (0.65-1.78)
LODDS, median [IQR]	-2.15 [-2.42, -1.10]	1.335 (1.016-1.754)
TC, median [IQR]	6.02 [4.46, 9.05]	0.999 (0.995-1.002)
TG, median [IQR]	2.13 [1.36, 3.62]	0.998 (0.994-1.001)
HDL, median [IQR]	0.850 [0.42, 1.47]	0.979 (0.951-1.007)
LDL, median [IQR]	3.17 [2.10, 4.74]	0.993 (0.983-1.003)
CA19-9, median [IQR]	61.5 [18.99, 194.18]	1.000 (1.000, 1.000)^*^
CEA, median [IQR]	2.68 [1.84, 3.80]	0.998 (0.995-1.002)

LODDS, log odds of positive lymph nodes; TC, total cholesterol; TG, triglyceride; HDL, high-density lipoprotein cholesterol; LDL, low-density lipoprotein cholesterol; CA19-9, carbohydrate antigen 19-9; CEA, carcinoembryonic antigen; RR, relative risk; AJCC, american joint committee on cancer staging manual; NA, not applicable.

^*^The lower bound is slightly below 1, while the upper bound is slightly above 1.

In the Western cohort, a total of 8128 patients with ampullary adenocarcinoma were identified from 2000 to 2019. After excluding patients with other pathological types (n = 2816), non-curative surgery (n = 1069), adjuvant therapy other than ACT (n = 348), distant metastasis (n = 314), and missing pathological data and follow-up information (n = 2633), 948 patients (mean [SD] age, 65.4 [11.0] years, 546 males [57.6%]) (**Figure 1B**). The major participants in the cohort tended to be elder (≥65 years, 523 [55.2%]), staged T3 (257 [27.1%]), staged N0 (457 [48.2%]), sized 2 to 4 cm (451 [47.6%]), moderately differentiated (521 [55.0%]), the White (715 [75.4%]), receiving no chemotherapy (610 [64.3%]), and married (591 [62.3%]) (**Supplementary Table 1**).

### 3.2 Survival analysis of ACT in total unselected patients

In the China cohort, the 5-year OS rates were 48.1% and 46.3% for the ACT group and non-ACT group, respectively (ACT HR, 0.96, 95% CI, 0.60 - 1.54, *P* = 0.86) (**Supplementary Figure 1A**). There was also no significant difference in RFS between the two groups (5-year RFS rate, 44.2% *vs* 45.8%; ACT HR: 1.05, 95% CI: 0.66 - 1.69, *P* = 0.84) (**Supplementary Figure 1B**). The LODDS and N classification were identified as multicollinearities, and the latter was excluded from the Cox regression model. Independent prognostic factors for decreased OS in patients with AA after curative resection were T stage (T3–4 *vs* T1-2, HR: 1.85, 95% CI, 1.21-2.81, *P* = 0.004) and increasing LODDS (HR: 1.51, 95% CI, 1.20-1.91, *P* < 0.001) (**Table 2**). Risk factors of decreased RFS were also identified as T stage (T3–4 *vs* T1-2, HR: 1.73, 95% CI, 1.13-2.64, *P* = 0.011) and increasing LODDS (HR: 1.55, 95% CI, 1.22-1.96, *P* < 0.001) (**Supplementary Table 2**).

**Table 2:** Univariate and multivariate cox proportional hazards regression identifying prognostic factors of overall survival in the China cohort.

Characteristics	Univariable		Multivariable	
	HR (95%CI)	P-value	HR (95%CI)	P-value
Sex				
Male	1 [Reference]	NA	NA	NA
Female	0.85 (0.57-1.25)	0.411	NA	NA
Age				
<45	1 [Reference]	NA	NA	NA
45-55	0.99 (0.49-1.99)	0.978	NA	NA
55-65	1.43 (0.73-2.78)	0.297	NA	NA
≥65	1.28 (0.63-2.60)	0.503	NA	NA
Complication				
No	1 [Reference]	NA	NA	NA
Yes	1.25 (0.85-1.84)	0.263	NA	NA
Blood transfusion				
No	1 [Reference]	NA	NA	NA
Yes	0.94 (0.64-1.38)	0.748	NA	NA
Size	1.04 (0.86-1.25)	0.681	NA	NA
Grade				
Poorly differentiated; Grade III	1 [Reference]	NA	1 [Reference]	
Moderately differentiated; Grade II	0.89 (0.58-1.36)	0.595	1.02 (0.66-1.56)	0.942
Well differentiated; Grade I	0.63 (0.37-1.08)	0.093	0.88 (0.50-1.57)	0.673
Lymphovascular invasion				
No	1 [Reference]	NA	NA	NA
Yes	1.38 (0.89-2.13)	0.154	NA	NA
AJCC 8th pT				
T1-2	1 [Reference]	NA	1 [Reference]	
T3-4	2.11 (1.42-3.13)	<0.001	1.85 (1.21-2.81)	0.004
LODDS	1.59 (1.27-1.98)	<0.001	1.51 (1.20-1.91)	<0.001
Chemotherapy				
No/Unknown	1 [Reference]	NA	NA	NA
Yes	0.96 (0.60-1.54)	0.863	NA	NA

LODDS, log odds of positive lymph nodes; HR, hazard ratio; AJCC, american joint committee on cancer staging manual; NA, not applicable.

In the Western cohort, no significant difference in OS was found between ACT and non-ACT groups (5-year OS rate, 40.9% *vs* 43.0%; ACT HR: 0.87, 95% CI: 0.73 - 1.03, *P* = 0.11) (**Supplementary Figure 1C**). The 5-year CSS rates were 46.0% and 53.1% for the two groups, respectively (ACT HR, 1.02, 95% CI, 0.84 - 1.24, *P* = 0.82) (**Supplementary Figure 1D**). The N classification was excluded from the Cox regression model due to multicollinearity. Independent predictive factors for decreased OS included ≥65 years (*P* = 0.014), T classification (T3–4 *vs* T1-2, *P* < 0.001), grade (III-IV *vs* I-II, *P* = 0.008), and increasing LODDS (*P* < 0.001), while factors for increasing OS were the White (*P* = 0.004) and annual income > 75,000$ (*P* = 0.041) (**Supplementary Table 3**). The prognostic factors for CSS were also identified using multivariate Cox regression, as shown in **Supplementary Table 4**.

### 3.3 Optimal LODDS for stratification and benefit analysis in selected patients

**Figure 2:**
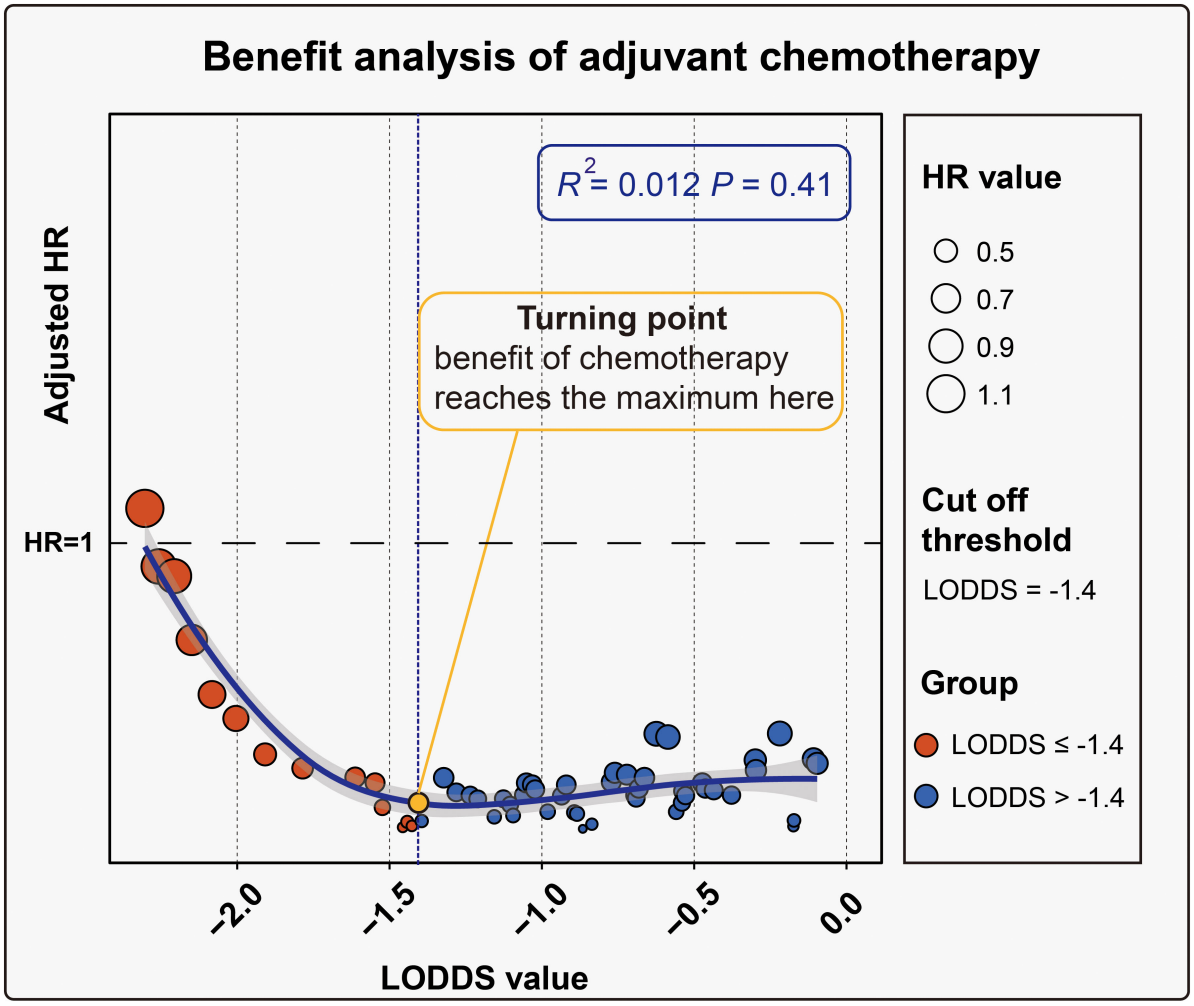
Identification of the optimal LODDS for stratification. The analysis was performed based on the trend between LODDS and adjusted HR; The value of HR was reflected by the shape area.

**Figure 3:**
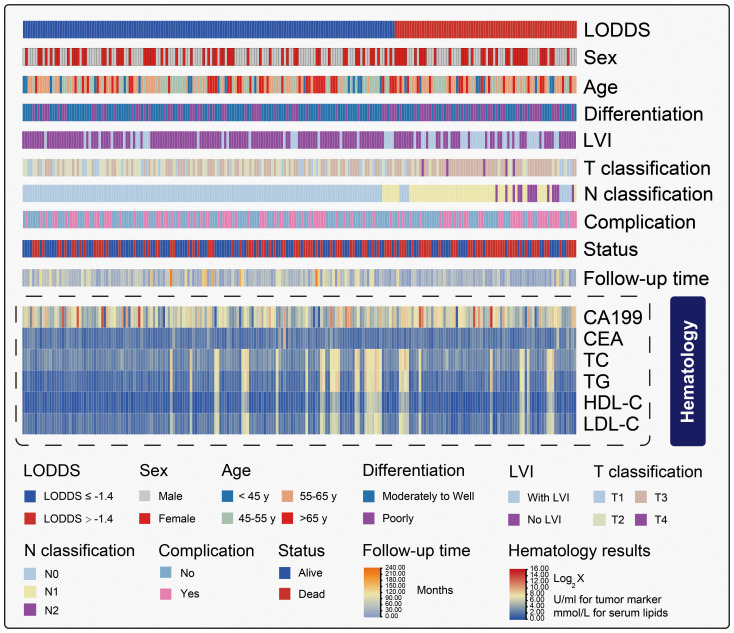
Clinicopathologic heatmap of AA patients in the China cohort stratified by LODDS. LVI, lymphovascular invasion; TC, total cholesterol; TG, triglyceride; HDL, high-density lipoprotein cholesterol; LDL, low-density lipoprotein cholesterol; CA19-9, carbohydrate antigen 19-9; CEA, carcinoembryonic antigen. The hematology results were converted using a logarithmic transformation with a base of two.

To determine the optimal LODDS for identifying benefits from ACT, stratified analyses were performed at each LODDS value and the adjusted HRs were calculated (range: 1.10 - 0.30). Spearman test indicated a nonlinear correlation between LODDS and adjusted HR (R^2^ = 0.012, *P* = 0.41). After a smooth trendline was applied using the LOESS regression, we recognized that as the value of LODDS increased, the HR gradually decreased. However, once LODDS reached -1.4, the trend became stable, suggesting that -1.4 was the optimal LODDS for patient stratification (**Figure 2**). Then, the patients were divided into the high-risk group (LODDS>-1.4) and the low-risk group (LODDS≤-1.4). Pearson χ^2^ test was used to examine the ACT regimen-related bias, indicating that no significant difference was identified in the distributions of gemcitabine-based and fluorouracil/other-based regimens in the two groups (*P* = 0.40, χ^2^ = .72) (**Supplementary Table 5**). We also demonstrated baseline comparison between the LODDS > -1.4 and LODDS ≤ -1.4 groups in **Supplementary Table 6**. Additionally, the distributions of LODDS were balanced in the two regimen groups as well (fluorouracil/other-based *vs* gemcitabine-based, mean [standard deviation, SD], -1.60 [0.92] *vs* -1.54 [0.81], *P* = 0.81). To obtain a comprehensive view of the characteristics, we depicted the clinicopathologic heatmap of AA patients in the China cohort, which was stratified by LODDS (**Figure 3**).

**Figure 4:**
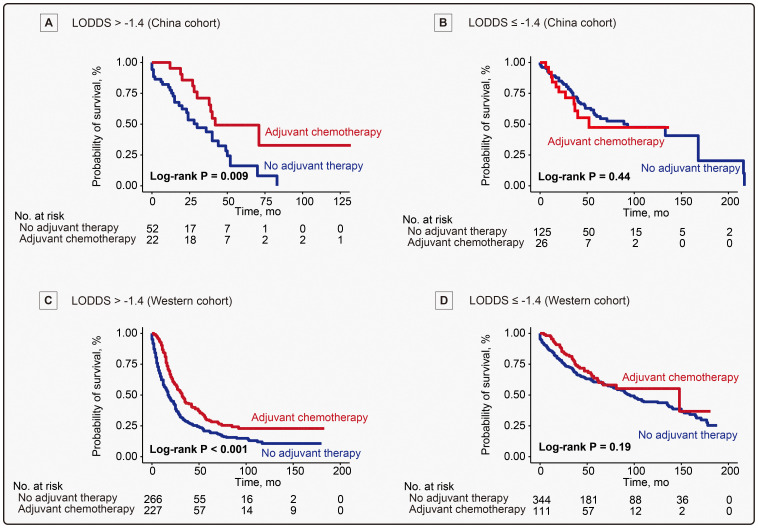
Kaplan-meier analysis evaluating influence of adjuvant chemotherapy on overall survival in patients with or without LODDS exceeding -1.4. **(A)** Overall survival (OS) of 74 patients with LODDS > -1.4, with or without adjuvant chemotherapy (ACT) in the China cohort. **(B)** OS of 151 patients with LODDS no more than -1.4 in the China cohort. **(C)** OS of 493 patients with LODDS > -1.4 in the Western cohort. **(D)** OS of 455 patients with LODDS no more than -1.4 in the Western cohort.

In AA patients with LODDS exceeding -1.4, the survival distribution stratified by the use of ACT revealed a significant difference for OS in the China cohort (5-year OS rate, 49.2% *vs* 16.2%, ACT HR, 0.40, 95% CI, 0.20 - 0.81, *P* = 0.01) and the Western cohort (5-year OS rate, 29.8% *vs* 20.8%, ACT HR, 0.61, 95% CI, 0.50 - 0.75, *P* < 0.001) (**Figures 4A, C**). Further analysis indicated that a potential difference was found in RFS of China cohort (*P* = 0.09), while a significant difference emerged in CSS of the Western cohort (*P* < 0.001) (**Supplementary Figures 2A, C**). Regarding patients with LODDS no more than -1.4, the analysis demonstrated that there was no significant difference between ACT and non-ACT groups for OS (*P* = 0.44) (**Figure 4B**) and RFS (*P* = 0.50) (**Supplementary Figure 2B**) in the China cohort, and OS (*P* = 0.19) (**Figure 4D**) and CSS (*P* = 0.92) (**Supplementary Figure 2D**) in the Western cohort.

### 3.4 Subgroup and interaction analysis

**Figure 5:**
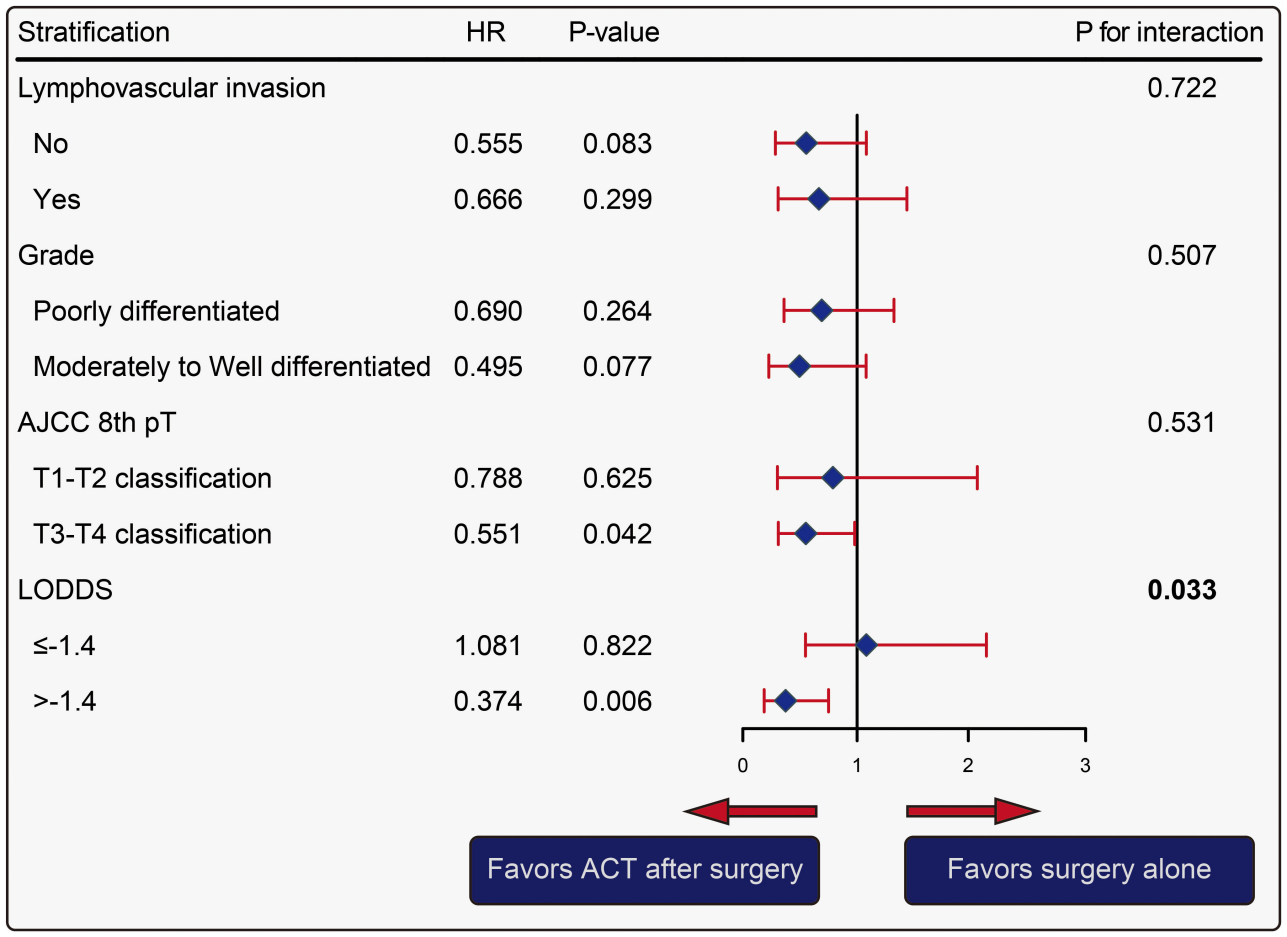
Forest plot of the effect of adjuvant chemotherapy versus surgery alone in patients with varying value of LODDS, adjusted by pathological factors. The analysis of LODDS was adjusted by lymphovascular invasion (LVI), differentiation (Grade), and pathologic T classification.

Stratified analysis of LODDS based on a multivariate Cox regression model was adjusted by pathological variables including LVI, grade, and the T classification. Interaction analysis for OS indicated that the differential benefit of ACT in the two LODDS groups was significant (interaction *P* = 0.033), suggesting patients with LODDS exceeding -1.4 favored ACT after surgery (ACT HR, 0.37, 95% CI, 0.19 - 0.75, *P* = 0.006). The results indicate that the precision of ACT application could be enhanced, particularly in resource-constrained settings, by incorporating LODDS into standard pathological assessments. In the subgroup analysis of LVI and grade, there was no significant difference in prognosis between the two groups (all *P >*0.05). Although a significant difference in OS was found between the two groups in patients with T3–4 stages (HR, 0.55, 95% CI, 0.31 - 0.98, *P* = 0.042), the interaction analysis showed no significance (interaction *P* = 0.531) (**Figure 5**). Similarly, the multivariate analysis of the N classification adjusted by LVI, grade, and the T classification was further performed. Compared with surgery alone, patients with N1–2 stages (*P* = 0.018) or T3–4 stages (*P* = 0.037) tended to have improved OS by using ACT. However, the interaction test demonstrated that the differential benefit was insignificant for the N classification (interaction *P* = 0.200) and the T classification (interaction *P* = 0.560) (**Supplementary Figure 3**). Patients stratified by LODDS demonstrated a more significant survival benefit from ACT compared to those stratified by N-staging.

## 4 Discussion

ampullary adenocarcinoma, a rare malignancy, lacks standardized guidelines for effective multimodal treatment following curative resection in the NCCN, ESMO, and JSHBPS (8, 10, 11). This area remains poorly studied, with comparably scant research conducted on it. The application pattern of ACT relies on extrapolations from the guidelines for nearby periampullary malignancies or the clinical experience of oncologists. In this cohort study, a large scale of sample size was conducted by drawing on the collective experience of the National Cancer Center of China and 12 registries from the West. The patients treated with radiotherapy were excluded to concentrate on the effect of ACT. Mainly focused issues of ACT addressed in the study are as follows: 1) the role of ACT in improving long-term survival for patients with AA after curative resection. 2) the role of LODDS in identifying postoperative AA patients benefiting from ACT. 3) compared with T and N classifications reported previously, the advantage of LODDS in identifying ACT-benefited patients.

At the beginning of the study, we performed a survival analysis stratified by the use of ACT in total enrolled patients. It suggested that compared with surgery alone, ACT was not associated with improved long-term survival of AA patients both in the China cohort and the Western cohort. These findings are corroborated by a meta-analysis including 1671 patients in fourteen studies, which found no associated survival benefit for ACT or combined with radiotherapy in the periampullary malignancy (14). Additionally, a multi-institution, international study reached similar results that neither intestinal nor pancreatobiliary histologic subtypes of AA treated with ACT demonstrated any significant improvement in survival, regardless of the chemotherapeutic regimen utilized (15). It is noteworthy that although ACT fails to prolong survival in all AA patients, the clinic experience from Mayo and other centers showed that ACT improved the survival of selected AA patients with pT3–4 or pN1-2 (9, 16). The findings provided enlightenment that burden of invasion and lymph node may be useful factors for identifying ACT-benefited patients. pN is a qualitative variable presenting lymph node burden, while LODDS is a quantitative variable that can continuously and accurately reflect the burden (12). We herein perform the study to determine the role of LODDS in selecting patients benefiting from ACT.

After evaluating the optimal value of LODDS for stratified analysis, -1.4 was identified. Patients with LODDS greater than or no more than -1.4 were divided into the high-risk group and the low-risk group, respectively. In the high-risk group, the use of ACT was significantly associated with improved long-term survival in the China cohort and the Western cohort. However, in the low-risk group, no significance in ACT-induced survival benefit was observed in the two cohorts. The findings indicated that AA patients with a larger value of LODDS (heavier burden of lymph node involvement) could benefit from ACT. The enhancement in survival rates among LODDS high-risk patients receiving ACT aligns well with the well-established benefits of adjuvant therapy in the setting of other gastrointestinal (GI) malignancies. For instance, the resected gastric cancer intergroup trial (INT0116), a randomized trial focusing on malignancies of the upper GI tract, demonstrated a notably improved survival after using adjuvant therapy in patients with regional lymph node involvement (17). The report of a previous study found that patients with resected AA may benefit from gemcitabine-based ACT, especially those with pancreatobiliary and/or mixed subtypes (18). Considering the influence of chemotherapy regimens on the survival benefit of AA, we performed a test of the regimen distributions in high-risk and low-risk patients, which showed a balanced regimen selection in the two patient groups. Therefore, the predictive role of LODDS in the benefit from ACT was determined.

Although previous studies demonstrated that pT3–4 and pN1–2 could identify the survival benefit from ACT (9, 16), the limitation should be addressed that due to the specificity as qualitative variables, the T and N classifications cannot predict the increasing effectiveness of ACT through continuous incremental changes. We further performed a comparison of the benefit-predictive role between LODDS and the stages of T and N. The interaction analysis showed that the differential benefit was significant across groups divided by LODDS, while no significance was found between groups divided by T or N classifications. The findings indicate the better ability of LODDS to identify ACT beneficiaries, suggesting that LODDS is probably associated with ACT benefit in a manner similar to the dose-response way. Regarding the quantitative factors for identifying benefits from ACT, similar studies were also conducted to evaluate the roles of examined lymph node (ELN) and positive lymph node (PLN) in lung cancer, and head and neck cancer, respectively (19, 20). However, the reliability of ELN and PLN in evaluating nodal involvement of GI malignancies has come into question (21). For example, the PLN is highly dependent on the ELN (22). The extent of lymphadenectomy during resection for adenocarcinoma, as well as the average number of ELN during surgery, both vary widely. Additionally, node-negative patients do not represent that there is no burden of lymph node involvement. As a ratio that comprehensively considers ELN, PLN, and node-negative patients, LODDS to some extent solves the above problems. In addition, the utility of LODDS has also been demonstrated in other gastrointestinal cancers, suggesting its potential as a universal metric for lymph node involvement (21, 23, 24). Given these considerations, the advantage of LODDS in predicting the survival benefits of ACT in AA patients is prominently emphasized.

The study has several limitations, including its retrospective nature and the potential bias of patient selection. All the participants from the National Cancer Center of China and the Western cohort were retrospectively reviewed. The results of this retrospective study may be influenced by selection bias and unmeasured confounding. In addition, the subtypes of intestinal and pancreatobiliary AA were not classified in the two cohorts. It is generally accepted that the major difference among these two subtypes is the status of nodal involvement (8). Adjuvant chemotherapy protocols encompass a diverse range of regimens, each tailored to specific clinical contexts and patient profiles. This diversity is essential for optimizing treatment outcomes, as it allows for personalized approaches that can better address the unique characteristics of individual cases. However, it also introduces a layer of complexity, making it challenging to draw broad, one-size-fits-all conclusions. To address this issue, we test the distribution of LODDS in the China cohort, which reflect that the node status between the two regimen groups is balanced (fluorouracil/other-based *vs* gemcitabine-based, *P* = 0.81). However, the test cannot be performed in the Western cohort due to the unknown information of regimens. Moreover, the study did not make a preplan of sample size and ACT performance, as well as a power analysis, which constrained our ability to assess the results. The results of this study are all retrospective, and more prospective studies and randomized controlled trials are needed before promoting LODDS risk stratification guided chemotherapy in clinical practice.

In summary, the findings of this cohort study indicate that the administration of ACT does not confer a survival advantage in an unselected cohort of patients with AA. However, a significant improvement in long-term survival was observed in patients with LODDS exceeding -1.4 who received ACT. These results underscore the clinical relevance of ACT in specific subpopulations of AA patients. The LODDS, serving as a reliable quantitative measure of nodal involvement, aids in the decision-making process regarding the application of ACT for AA patients post-curative surgery. These findings substantiate the incorporation of LODDS into clinical guidelines for ACT decision-making in AA. Additionally, this metric has the potential to serve as a foundation for future personalized treatment algorithms. Prospective multicenter studies are essential to confirm these findings and facilitate their translation into clinical practice.

## Data Availability

The data analyzed in this study is subject to the following licenses/restrictions: The datasets used during the current study are available from the corresponding author upon reasonable request without ethical, privacy, or security concerns. Requests to access these datasets should be directed to drlizheng1008@126.com.
