# Supplementary material for: Quantitative nodal burden as a predictive marker for chemotherapy benefit in postoperative ampullary adenocarcinoma: a multi-institution population-based study
Source: Front Immunol. 2025 Sep 19;16:1610334. doi: 10.3389/fimmu.2025.1610334 (PMC12491244; doi:10.3389/fimmu.2025.1610334)
Supplement: Supplementary file 1 [file DataSheet1.docx]

Supplementary Material

## Supplementary Figures

**
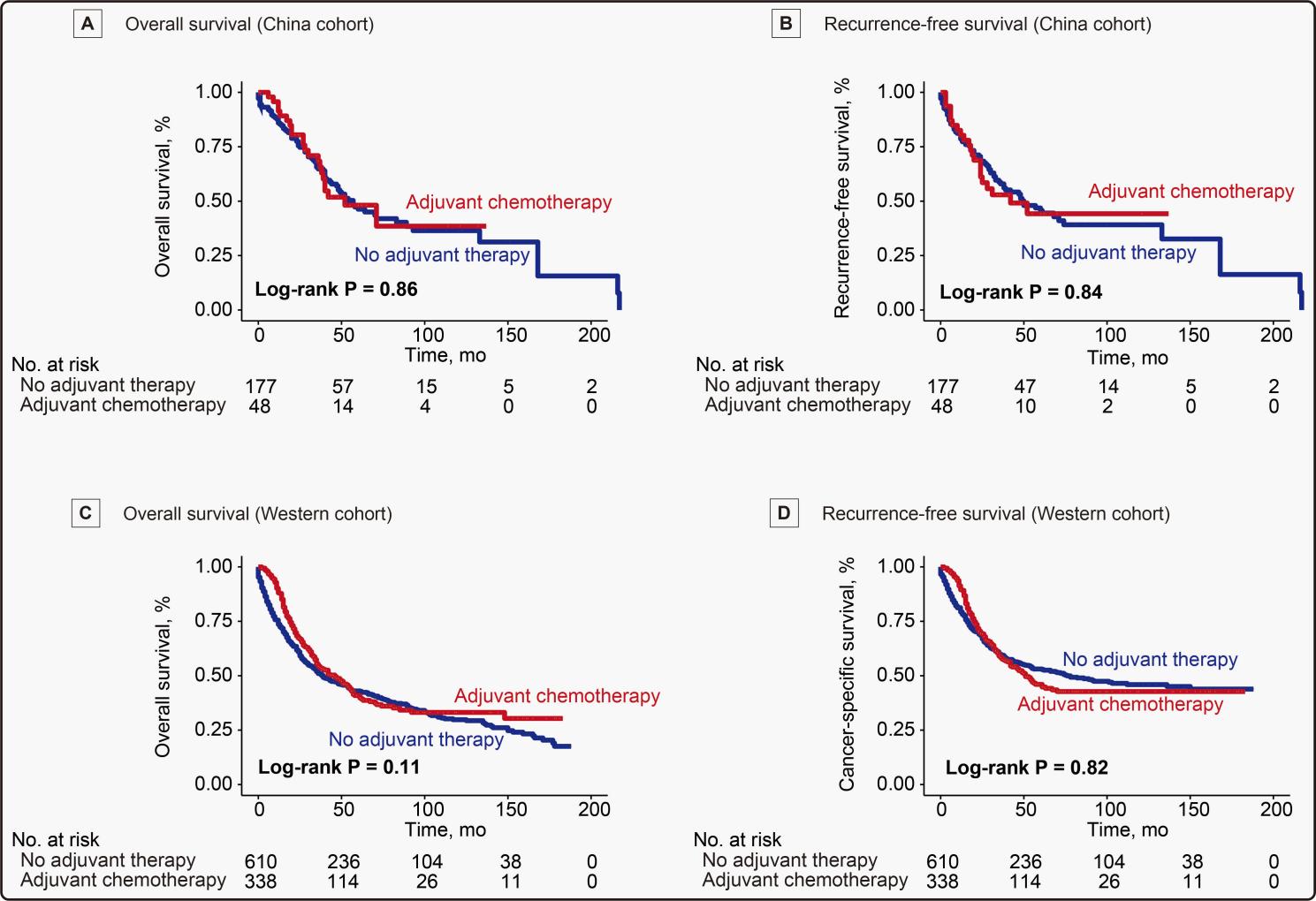
**

**Supplementary Figure 1.** Kaplan-Meier Estimates of Overall Survival, Recurrence-Free Survival, and Cancer-Specific Survival, Stratified by Receipt of Adjuvant Chemotherapy.

A, Overall survival of 225 patients with or without adjuvant chemotherapy (ACT) in the China cohort. B, Recurrence-free survival of the patients in the China cohort. C, Overall survival of 948 patients with or without ACT in the Western cohort. D, Cancer-specific survival of the patients in the Western cohort.


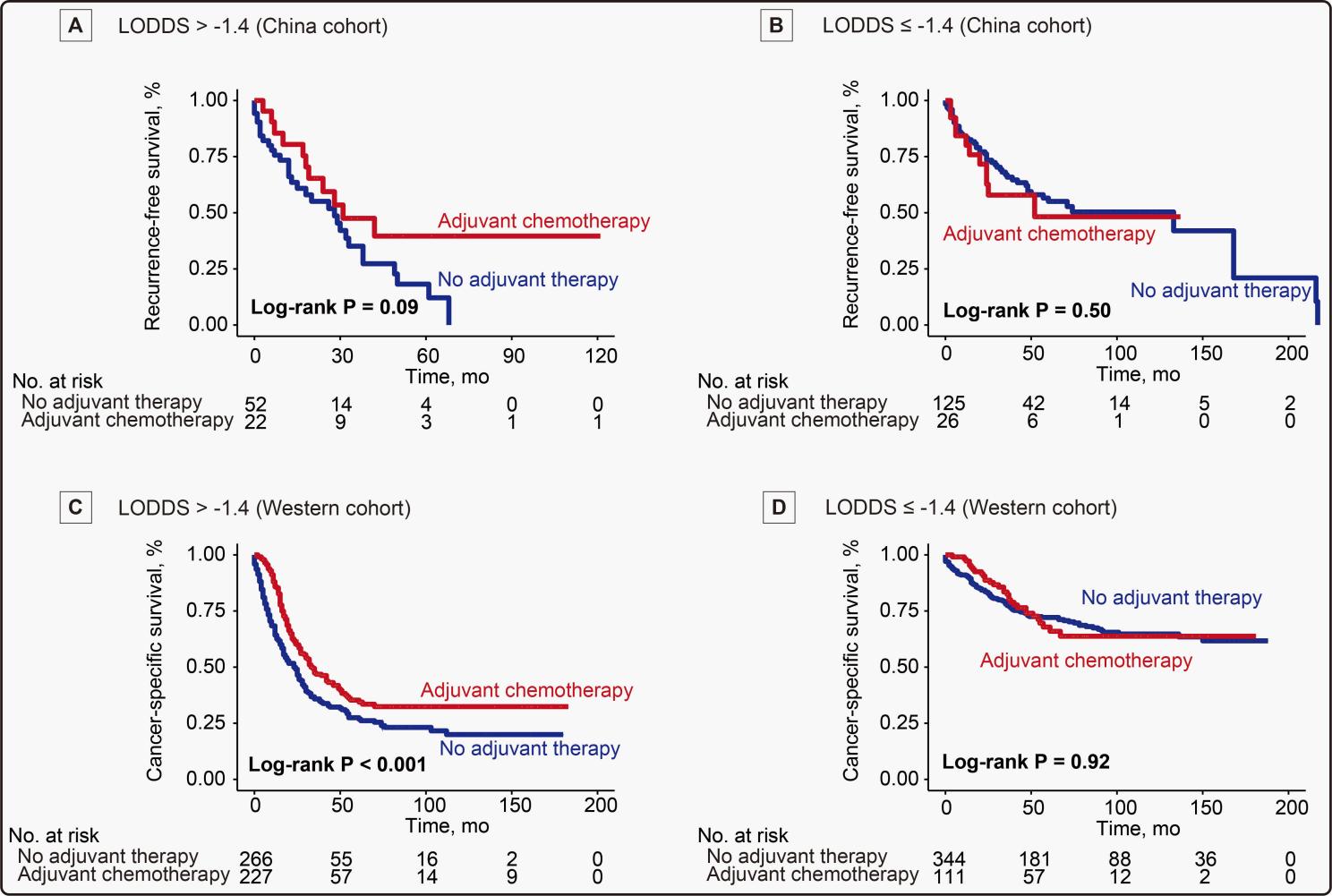


**Supplementary Figure 2**. Kaplan-Meier Estimates Evaluating Effect of Adjuvant Chemotherapy on Recurrence-Free Survival and Cancer-Specific Survival in Patients With or Without LODDS exceeding -1.4.

A, Recurrence-free survival (RFS) of 74 patients with LODDS > -1.4, with or without adjuvant chemotherapy (ACT) in the China cohort. B, RFS of 151 patients with LODDS no more than -1.4 in the China cohort. C, Cancer-specific survival (CSS) of 493 patients with LODDS > -1.4 in the Western cohort. D, CSS of 455 patients with LODDS no more than -1.4 in the Western cohort.


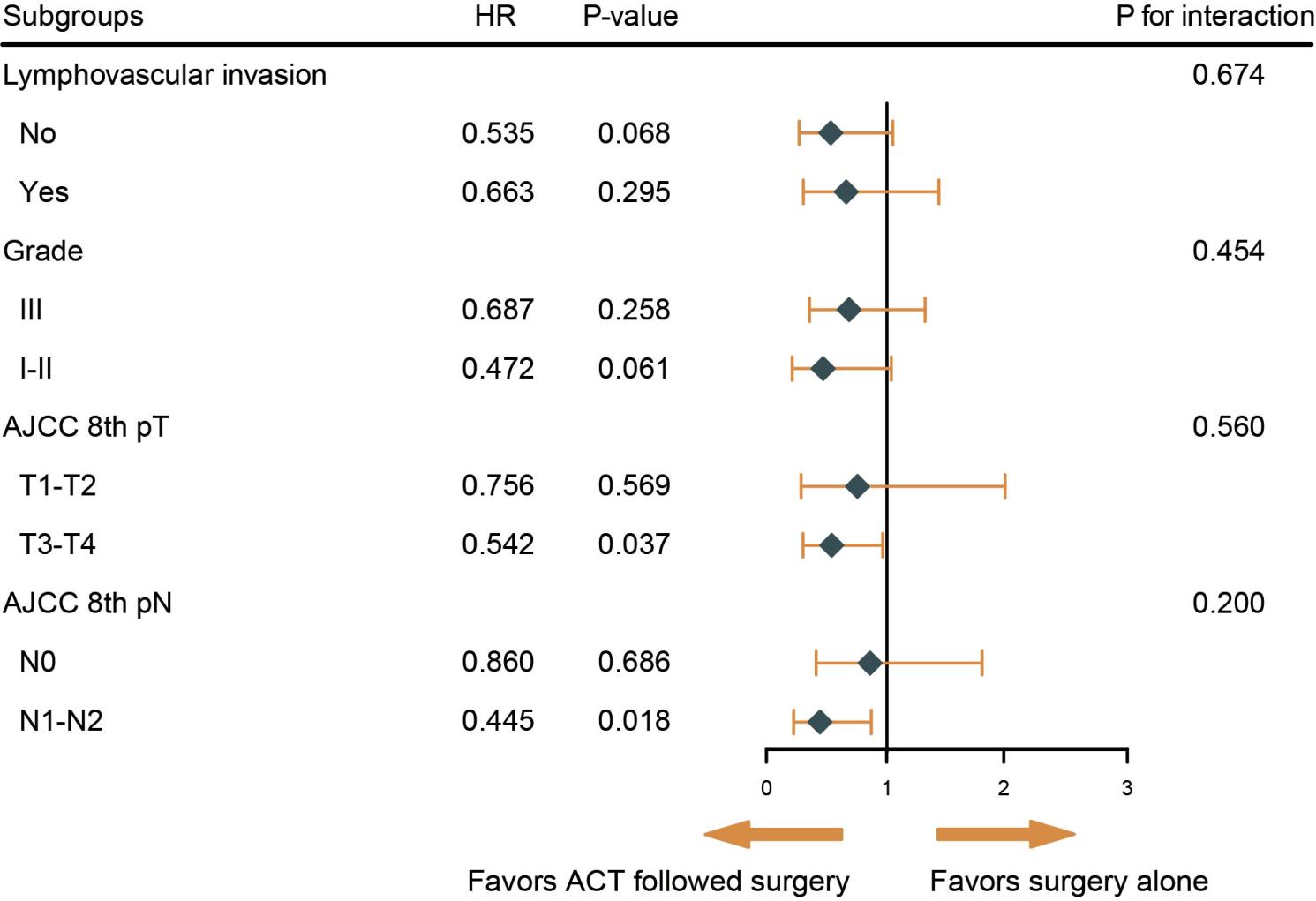


**Supplementary Figure 3.** Forest Plot of the Effect of Adjuvant Chemotherapy Versus Surgery Alone in Patients with Varying N and T classifications, Adjusted by Pathological Factors.

The analysis of pathologic T and N classifications was adjusted by lymphovascular invasion (LVI), differentiation (Grade), and each other.

## Supplementary Tables

**Supplementary Table 1.** Baseline Characteristics of Demographic, Clinicopathologic, and Outcome of the Western cohort.

| Variable | Value (N=948) |  |
| --- | --- | --- |
|  |  |  |
| Sex, n (%) |  |  |
| Male | 546 (57.6%) |  |
| Female | 402 (42.4%) |  |
| Age, n (%) |  |  |
| <45 | 35 (3.7%) |  |
| 45-55 | 121 (12.8%) |  |
| 55-65 | 269 (28.4%) |  |
| ≥65 | 523 (55.2%) |  |
| AJCC 8th pT, n (%) |  |  |
| T1 | 96 (10.1%) |  |
| T2 | 213 (22.5%) |  |
| T3 | 257 (27.1%) |  |
| T4 | 234 (24.7%) |  |
| NA | 148 (15.6%) |  |
| AJCC 8th pN, n (%) |  |  |
| N0 | 457 (48.2%) |  |
| N1 | 305 (32.2%) |  |
| N2 | 186 (19.6%) |  |
| Size, n (%) |  |  |
| <2cm | 373 (39.3%) |  |
| 2-4cm | 451 (47.6%) |  |
| ≥4cm | 124 (13.1%) |  |
| Grade, n (%) |  |  |
| Undifferentiated; anaplastic; Grade IV | 11 (1.2%) |  |
| Poorly differentiated; Grade III | 306 (32.3%) |  |
| Moderately differentiated; Grade II | 521 (55.0%) |  |
| Well differentiated; Grade I | 110 (11.6%) |  |
| Race, n (%) |  |  |
| Black | 75 (7.9%) |  |
| Other (American Indian/AK Native, Asian/Pacific Islander,Unknown) | 158 (16.7%) |  |
| White | 715 (75.4%) |  |
| Chemotherapy, n (%) |  |  |
| No/Unknown | 610 (64.3%) |  |
| Yes | 338 (35.7%) |  |
| Months from diagnosis to treatment, n (%) |  |  |
| 0-1 | 388 (40.9%) |  |
| 1-2 | 383 (40.4%) |  |
| ≥2 | 177 (18.7%) |  |
| Income, n (%) |  |  |
| <55,000$ | 155 (16.4%) |  |
| 55,000-75,000$ | 505 (53.3%) |  |
| >75,000$ | 288 (30.4%) |  |
| Marital status at diagnosis, n (%) |  |  |
| Married (including common law) | 591 (62.3%) |  |
| Not married | 357 (37.7%) |  |
| LODDS, median [IQR] | -1.32 [-2.38, -0.65] |  |

**Supplementary Table 2.** Univariate and Multivariate Cox Proportional Hazards Regression Identifying Prognostic Factors of Recurrence-Free Survival in the China cohort.

|  | Univariable | |  | Multivariable | |
| --- | --- | --- | --- | --- | --- |
| Characteristics | HR (95%CI) | P-value |  | HR (95%CI) | P-value |
| Sex |  |  |  |  |  |
| Male | 1 [Reference] | NA |  | NA | NA |
| Female | 0.9 (0.61-1.34) | 0.612 |  | NA | NA |
| Age |  |  |  |  |  |
| <45 | 1 [Reference] | NA |  | NA | NA |
| 45-55 | 0.98 (0.49-1.97) | 0.959 |  | NA | NA |
| 55-65 | 1.46 (0.75-2.85) | 0.267 |  | NA | NA |
| ≥65 | 1.19 (0.58-2.42) | 0.638 |  | NA | NA |
| Complication |  |  |  |  |  |
| No | 1 [Reference] | NA |  | NA | NA |
| Yes | 1.24 (0.84-1.82) | 0.28 |  | NA | NA |
| Blood transfusion |  |  |  |  |  |
| No | 1 [Reference] | NA |  | NA | NA |
| Yes | 0.98 (0.67-1.44) | 0.927 |  | NA | NA |
| Size | 1.07 (0.89-1.3) | 0.46 |  | NA | NA |
| Grade |  |  |  |  |  |
| Poorly differentiated; Grade III | 1 [Reference] | NA |  | 1 [Reference] |  |
| Moderately differentiated; Grade II | 0.92 (0.6-1.41) | 0.697 |  | 1.09(0.71-1.67) | 0.703 |
| Well differentiated; Grade I | 0.61 (0.35-1.05) | 0.073 |  | 0.85(0.48-1.52) | 0.591 |
| Lymphovascular invasion |  |  |  |  |  |
| No | 1 [Reference] | NA |  | NA | NA |
| Yes | 1.43 (0.92-2.23) | 0.107 |  | NA | NA |
| AJCC 8th pT |  |  |  |  |  |
| T1-2 | 1 [Reference] | NA |  | 1 [Reference] |  |
| T3-4 | 2.07 (1.39-3.06) | <0.001 |  | 1.73(1.13-2.64) | 0.011 |
| LODDS | 1.64 (1.31-2.05) | <0.001 |  | 1.55(1.22-1.96) | <0.001 |
| Chemotherapy |  |  |  |  |  |
| No/Unknown | 1 [Reference] | NA |  | NA | NA |
| Yes | 1.05 (0.66-1.69) | 0.834 |  | NA | NA |

Abbreviations: NA, not applicable

**Supplementary Table 3.** Univariate and Multivariate Cox Proportional Hazards Regression Identifying Prognostic Factors of Overall Survival in the Western cohort.

|  | Univariable | |  | Multivariable | |
| --- | --- | --- | --- | --- | --- |
| Characteristics | HR (95%CI) | P-value |  | HR (95%CI) | P-value |
| Sex |  |  |  |  |  |
| Female | 1 [Reference] | NA |  | NA | NA |
| Male | 1.07(0.91-1.26) | 0.43 |  | NA | NA |
| Age |  |  |  |  |  |
| <45 | 1 [Reference] | NA |  | 1 [Reference] | NA |
| 45-55 | 1.1(0.63-1.9) | 0.745 |  | 1.15(0.66-2.02) | 0.614 |
| 55-65 | 1.38(0.83-2.31) | 0.217 |  | 1.46(0.87-2.45) | 0.155 |
| ≥65 | 1.74(1.06-2.88) | 0.03 |  | 1.88(1.13-3.13) | 0.014 |
| Race |  |  |  |  |  |
| Black | 1 [Reference] |  |  | 1 [Reference] | NA |
| White | 0.67(0.5-0.89) | 0.005 |  | 0.62(0.45-0.86) | 0.004 |
| Other | 0.68(0.49-0.96) | 0.026 |  | 0.74(0.51-1.08) | 0.114 |
| Income |  |  |  |  |  |
| <55,000$ | 1 [Reference] |  |  | 1 [Reference] | NA |
| 55,000-75,000$ | 0.79(0.63-0.98) | 0.033 |  | 0.81(0.64-1.02) | 0.076 |
| >75,000$ | 0.7(0.55-0.89) | 0.004 |  | 0.76(0.58-0.99) | 0.041 |
| Size |  |  |  |  |  |
| <2cm | 1 [Reference] |  |  | 1 [Reference] | NA |
| 2-4cm | 1.5(1.26-1.79) | <0.001 |  | 1.13(0.92-1.37) | 0.241 |
| ≥4cm | 1.62(1.26-2.08) | <0.001 |  | 1.19(0.9-1.57) | 0.232 |
| Grade |  |  |  |  |  |
| Grade I-II | 1 [Reference] | NA |  | 1 [Reference] | NA |
| Grade III-IV | 1.47(1.25-1.74) | <0.001 |  | 1.29(1.07-1.55) | 0.008 |
| AJCC 8th pT |  |  |  |  |  |
| T1-2 | 1 [Reference] | NA |  | 1 [Reference] | NA |
| T3-4 | 2.01(1.67-2.43) | <0.001 |  | 1.47(1.2-1.79) | <0.001 |
| LODDS | 1.79(1.65-1.95) | <0.001 |  | 1.61(1.46-1.78) | <0.001 |
| Chemotherapy |  |  |  |  |  |
| No/Unknown | 1 [Reference] | NA |  | NA | NA |
| Yes | 0.87(0.73-1.03) | 0.113 |  | NA | NA |

Abbreviations: NA, not applicable

**Supplementary Table 4.** Univariate and Multivariate Cox Proportional Hazards Regression Identifying Prognostic Factors of Cancer-Specific Survival in the Western cohort.

|  | Univariable | |  | Multivariable | |
| --- | --- | --- | --- | --- | --- |
| Characteristics | HR (95%CI) | P-value |  | HR (95%CI) | P-value |
| Sex |  |  |  |  |  |
| Female | 1 [Reference] | NA |  | NA | NA |
| Male | 0.98(0.81-1.18) | 0.843 |  | NA | NA |
| Age |  |  |  |  |  |
| <45 | 1 [Reference] | NA |  | NA | NA |
| 45-55 | 0.87(0.49-1.52) | 0.617 |  | NA | NA |
| 55-65 | 1.12(0.67-1.89) | 0.666 |  | NA | NA |
| ≥65 | 1.19(0.72-1.98) | 0.492 |  | NA | NA |
| Race |  |  |  |  |  |
| Black | 1 [Reference] | NA |  | 1 [Reference] | NA |
| White | 0.63(0.46-0.87) | 0.005 |  | 0.65(0.45-0.93) | 0.019 |
| Other | 0.65(0.45-0.95) | 0.026 |  | 0.77(0.5-1.18) | 0.227 |
| Income |  |  |  |  |  |
| <55,000$ | 1 [Reference] | NA |  | 1 [Reference] | NA |
| 55,000-75,000$ | 0.77(0.6-0.99) | 0.042 |  | 0.76(0.58-0.99) | 0.041 |
| >75,000$ | 0.69(0.52-0.91) | 0.008 |  | 0.76(0.56-1.02) | 0.071 |
| Size |  |  |  |  |  |
| <2cm | 1 [Reference] | NA |  | 1 [Reference] | NA |
| 2-4cm | 1.51(1.23-1.85) | <0.001 |  | 1.01(0.81-1.28) | 0.909 |
| ≥4cm | 1.61(1.2-2.15) | 0.001 |  | 0.94(0.68-1.3) | 0.708 |
| Grade |  |  |  |  |  |
| Grade I-II | 1 [Reference] | NA |  | 1 [Reference] | NA |
| Grade III-IV | 1.59(1.31-1.92) | <0.001 |  | 1.28(1.03-1.59) | 0.023 |
| AJCC 8th pT |  |  |  |  |  |
| T1+T2 | 1 [Reference] | NA |  | 1 [Reference] | NA |
| T3+T4 | 2.28(1.82-2.86) | <0.001 |  | 1.59(1.25-2.01) | <0.001 |
| LODDS | 2.01(1.82-2.22) | <0.001 |  | 1.83(1.64-2.05) | <0.001 |
| Chemotherapy |  |  |  |  |  |
| No/Unknown | 1 [Reference] | NA |  | NA | NA |
| Yes | 1.02(0.84-1.24) | 0.821 |  | NA | NA |

Abbreviations: NA, not applicable

**Supplementary Table 5.** The distribution of chemotherapy regimens in the China cohort.

| Group | LODDS≤-1.4 | LODDS>-1.4 | P-value (χ^2^) |
| --- | --- | --- | --- |
| gemcitabine-based regimens | 11 | 12 | 0.40 (0.72) |
| fluorouracil/other-based regimens | 15 | 10 |  |

Pearson χ^2^ test was used to test the distribution.

**Supplementary Table 6.** The baseline characteristics of patients grouped by LODDS in the China cohort.

|  | LODDS<-1.4 | LODDS>-1.4 | P-value |
| --- | --- | --- | --- |
|  | (N=151) | (N=74) |  |
| **Age** |  |  |  |
| Mean (SD) | 56.1 (10.6) | 58.1 (9.38) | 0.158 |
| Median [Min, Max] | 56.0 [14.0, 78.0] | 59.0 [35.0, 77.0] |  |
| **Size** |  |  |  |
| Mean (SD) | 2.33 (0.990) | 2.57 (1.10) | 0.111 |
| Median [Min, Max] | 2.20 [0.500, 5.00] | 2.50 [1.00, 6.00] |  |
| **LVI** |  |  |  |
| No | 124 (82.1%) | 42 (56.8%) | <0.001 |
| Yes | 27 (17.9%) | 32 (43.2%) |  |
| **T classification** |  |  |  |
| T1+T2 | 85 (56.3%) | 18 (24.3%) | <0.001 |
| T3+T4 | 66 (43.7%) | 56 (75.7%) |  |
| **N classification** |  |  |  |
| N0 | 146 (96.7%) | 9 (12.2%) | <0.001 |
| N1+N2 | 5 (3.3%) | 65 (87.8%) |  |
